# Prenatal Exposure to Dietary Fat Induces Changes in the Transcriptional Factors,TEF and YAP, Which May Stimulate Differentiation of Peptide Neurons in Rat Hypothalamus

**DOI:** 10.1371/journal.pone.0077668

**Published:** 2013-10-11

**Authors:** Kinning Poon, Sushma Mandava, Karen Chen, Jessica R. Barson, Sylvie Buschlen, Sarah F. Leibowitz

**Affiliations:** The Rockefeller University, Laboratory of Behavioral Neurobiology, New York, New York, United States of America; CRCHUM-Montreal Diabetes Research Center, Canada

## Abstract

Gestational exposure to a high-fat diet (HFD) stimulates the differentiation of orexigenic peptide-expressing neurons in the hypothalamus of offspring. To examine possible mechanisms that mediate this phenomenon, this study investigated the transcriptional factor, transcription enhancer factor-1 (TEF), and co-activator, Yes-associated protein (YAP), which when inactivated stimulate neuronal differentiation. In rat embryos and postnatal offspring prenatally exposed to a HFD compared to chow, changes in hypothalamic TEF and YAP and their relationship to the orexigenic peptide, enkephalin (ENK), were measured. The HFD offspring at postnatal day 15 (P15) exhibited in the hypothalamic paraventricular nucleus a significant reduction in YAP mRNA and protein, and increased levels of inactive and total TEF protein, with no change in mRNA. Similarly, HFD-exposed embryos at embryonic day 19 (E19) showed in whole hypothalamus significantly decreased levels of YAP mRNA and protein and TEF mRNA, and increased levels of inactive TEF protein, suggesting that HFD inactivates TEF and YAP. This was accompanied by increased density and fluorescence intensity of ENK neurons. A close relationship between TEF and ENK was suggested by the finding that TEF co-localizes with this peptide in hypothalamic neurons and HFD reduced the density of TEF/ENK co-labeled neurons, even while the number and fluorescence intensity of single-labeled TEF neurons were increased. Increased YAP inactivity by HFD was further evidenced by a decrease in number and fluorescence intensity of YAP-containing neurons, although the density of YAP/ENK co-labeled neurons was unaltered. Genetic knockdown of TEF or YAP stimulated ENK expression in hypothalamic neurons, supporting a close relationship between these transcription factors and neuropeptide. These findings suggest that prenatal HFD exposure inactivates both hypothalamic TEF and YAP, by either decreasing their levels or increasing their inactive form, and that this contributes to the stimulatory effect of HFD on ENK expression and possibly the differentiation of ENK-expressing neurons.

## Introduction

Obesity is a growing epidemic, with the latest National Health and Nutrition Survey finding that 36% of adults and 17% of adolescents and children are obese [1]. Clinical and animal studies have attributed this rise, in part, to fetal programming produced by maternal obesity and overconsumption of a fat-rich diet, which in the offspring increases preference for a high-fat diet (HFD) and risk for higher weight gain and metabolic disorders [2-6]. The hypothalamus, which is an important part of the brain that controls energy homeostasis by regulating food intake and energy expenditure, has been shown to be markedly affected by prenatal exposure to a HFD. Maternal ingestion of a HFD stimulates neurogenesis in early embryonic hypothalamus and increases the number of neurons that express neuropeptides known to stimulate ingestive behavior [4,7]. Further, prenatal HFD exposure increases the peptide-expressing neuroprogenitor cell population in embryos, which later differentiate into functional peptide neurons [8,9]. The cellular factors involved in this stimulatory effect on the differentiation and expression of peptide neurons are unknown but are likely to involve transcriptional regulators of neuronal differentiation, many of which, with the exception of c-Fos and CREB [10-12], have never been studied in relation to the orexigenic peptides in the hypothalamus. 

The transcription enhancer factor-1 (TEF) is a transcriptional factor that partners predominantly with the transcriptional co-activator, Yes-associated protein (YAP) [13,14], and is very important for regulating organ growth during development, with genetic deletion of either TEF or YAP found to be fatal to the developing embryo [15,16]. The association of the active forms of TEF and YAP is found to induce transcriptional activation of cell-cycle related elements that affect neuronal differentiation and proliferation [17-19]. Phosphorylation of serine residues of these two transcriptional factors causes them to sequester in the cytoplasm and become inactive [20-23]. A suppression of TEF and YAP activity, either by decreasing levels or increasing the inactive forms of these proteins, activates neuronal differentiation while inhibiting proliferation, whereas excess TEF and YAP causes an expansion of neuroprogenitor cells by inhibiting differentiation and increasing proliferation [17,19,24]. Furthermore, genetic profiling of altered TEF or YAP activity has revealed changes in neuropeptide and neurotransmitter expression [25], hinting at the possibility that these two factors may be involved in the differentiation of hypothalamic neurons that express the orexigenic peptides and in the stimulatory effect produced by prenatal exposure to a HFD.

Recent studies focus attention on the orexigenic peptide, enkephalin (ENK), in terms of its close relationship to dietary fat in both adults and embryos. In adult rats, this opioid peptide stimulates the intake of a HFD when injected into the hypothalamic paraventricular nucleus (PVN) [26-28], and its levels are endogenously increased in the PVN of animals consuming or prone to overeating the HFD [29]. Also, exposure to a HFD during gestation is found to increase in the PVN not only the expression of ENK but also the number of ENK neurons in the offspring, as demonstrated both in postnatal day 15 (P15) offspring and embryonic day 19 (E19) embryos [4,9], an effect that is sustained into adulthood. As the hypothalamus forms early in development and undergoes restructuring at E19 [30,31] while becoming fully formed by P15, it is interesting that the expression of both TEF and YAP is also at its peak during the embryonic developmental period [32], while decreasing postnatally into adulthood [33]. 

This temporal overlap of neuronal differentiation with TEF and YAP expression suggests that changes in TEF and YAP activity may be contributing to the increase in differentiated ENK neurons and ENK expression that occurs in the offspring in response to maternal consumption of a HFD. Using various methods to quantify mRNA and protein levels in P15 offspring and E19 embryos, the present studies aimed to test whether prenatal HFD exposure affects TEF and YAP levels in the hypothalamus and whether TEF and YAP activity is related to the expression of ENK-expressing neurons. 

## Methods

### Postnatal rats

Timed-pregnant, Sprague-Dawley rats were acquired from Charles River Laboratories (Hartford, CT) on embryonic day 5 (E5). All experimental procedures were performed according to institutionally approved protocols as specified in the NIH Guide to the Care and Use of Animals and also with approval of the Rockefeller University Animal Care and Use Committee. The dams were individually housed in a fully accredited AAALAC facility (22°C, with a 12:12-h light-dark cycle with lights off at 12 pm). The rats were split into 2 groups of 8 dams and maintained *ad libitum* from E7 on either a high-fat diet (HFD; 5.02 kcal/g) with 50% fat or standard lab chow (3.36 kcal/g) with 13% fat (Purina, St. Louis, MO), as previously described [4,9,34]. Standard lab chow was available for 3 days (until E9) before complete removal, in order for the HFD group to overcome neophobia and adapt to the HFD. Over the course of the experiments, food intake was measured 3 times per week, and body weight was recorded weekly. There was no difference between the HFD and chow dams in their daily caloric intake during pregnancy (70-90 kcals) and lactation (100-125 kcals) or in their body weight at parturition (320-350 g). The litters of the HFD dams were similar to the chow litters in terms of size, body length, body weight and female/male ratio, with no spontaneous abortions observed in either diet group. On postnatal day 2 (P2), litters studied after birth were culled to n=8. Each experiment tested only male offspring, with 1 male pup taken from each litter (n=4-8). The HFD and chow offspring were sacrificed at P15 by rapid decapitation. Immediately after sacrifice, the postnatal brain was placed in a matrix with the ventral surface facing up, and one 1.0 mm coronal section was made, with the middle optic chiasm as the anterior boundary. The PVN was rapidly dissected at the level of bregma A3.8-3.5 mm under a microscope, using the fornix and third ventricle as landmarks and the stereotaxic atlas of a 10-day-old rat brain for guidance [35]. The PVN was dissected as a reversed isosceles triangle, 0.5 mm bilateral to the ventricle and between the fornix structures. The tissue was either placed in RNAlater (Invitrogen, Carlsbad, CA) for mRNA purification or immediately frozen in liquid nitrogen and stored at -80°C for western blot analysis.

### Embryonic rats

Timed-pregnant rats were given the same HFD paradigm as postnatal animals and sacrificed on embryonic day 19 (E19), as previously described [9]. The whole hypothalamus was extracted and dissociated for plating into cell culture or placed in either RNAlater for mRNA purification or immediately frozen in liquid nitrogen and stored at -80°C for western blot analysis. Whole hypothalamus was used since individual regions of the hypothalamus are not fully differentiated at this age. 

### Diets

The high-fat diet used in this report has been described in detail in previous publications [4,9,34]. The HFD consisted of 50% fat composed of 75% lard (Armour Star, Peoria, IL) and 25% vegetable oil (Crisco, Orrville, OH), 25% carbohydrate from 30% dextrin (ICN Pharmaceuticals, Costa Mesa, CA), 30% cornstarch (ICN Pharmaceuticals, Costa Mesa, CA), and 40% sucrose (Domino Foods Inc., Yonkers, NY), 25% protein from casein (Bio-Serv, Frenchtown, NJ), and was supplemented with minerals (USP XIV Salt Mixture Briggs; ICN Pharmaceuticals, Costa Mesa, CA) and vitamins (Vitamin Diet Fortification Mixture; ICN Pharmaceuticals, Costa Mesa, CA). This diet is nutritionally complete and does not have any detrimental effects on the health of the animals. 

### Cell culture

Hypothalami from E19 embryos were micro-dissected in Mg^2+^/Ca^2+^ free Hank’s balanced salt solution (Sigma-Aldrich, St. Louis, MO) and placed in 0.05% trypsin-EDTA for 30 min at 37°C (Invitrogen, Carlsbad, CA), as previously described [9]. The cells were triturated with 0.01% deoxyribonuclease in Neurobasal Media using a 1000 μL pipette tip, passed through a 70 µm followed by a 40 µm cell strainer (Fisher Scientific, Waltham, MA), and spun down. The cells (1 million / mL) were resuspended in Neurobasal Media containing B27 supplement (Invitrogen, Carlsbad, CA) and cultured at 1x10^6^ cells per well in a 6-well plate (BD Biosciences, Sparks, MD) or at 1x10^5^ cells on #1.5, 18 mm round coverslips (Warner Instruments, Hamden, CT). Cells were then placed in a humidified, 5% CO2 incubator at 35°C. All plates and coverslips were coated with poly-D-lysine (Sigma-Aldrich, St. Louis, MO) and rinsed with distilled water. Neurons were allowed to settle for 3 days for immunofluorescence imaging.

### Quantitative Polymerase Chain Reaction

The mRNA from each micro-dissected sample was purified using a Qiagen RNeasy kit (Qiagen, Valencia, CA), cDNA was synthesized using high capacity RNA-to-cDNA master mix (Applied Biosystems, Foster City, CA), and the SYBR Green PCR core reagents kit (Applied Biosystems, Foster City, CA) was used for qRT-PCR and was performed in MicroAmp Optic 96-well Reaction Plates (Applied Biosystems, Foster City, CA) under the condition of 2 min at 50°C, 10 min at 95°C, and 40 cycles of 15 s at 95°C and 1 min at 60°C, as previously described [9,36]. The levels of target gene expression were quantified relative to the level of cyclophilin-A, using the relative quantification method. Each sample was run in triplicate, and included a no-template control and a negative RT control. Primers were designed with the NCBI Primer design tool (http://www.ncbi.nlm.nih.gov/tools/primer-blast/) to span an exon-exon gap to eliminate amplification of genomic DNA. The primers used were: *TEF forward: 5′-CACAGCCCCCATTCCAGGGTTTG-3′, reverse: 5′-TTCGAGCAACGGGTCGCTGT-3′; YAP forward: 5’-CGTGCCCATGAGGCTTCGCA-3’, reverse: 5’-TCGGTACTGGCCTGTCGCGA-3’; ENK forward: 5'-GGACTGCGCTAAATGCAGCTA-3', reverse: 5'-GTGTGCATGCCAGGAAGTTG-3'; cyclophilin-A forward: 5′-GTGTTCTTCGACATCACGGCT-3′, reverse: 5′-CTGTCTTTGGAACTTTGTCTGCA-3′*. The specificities of PCR products were confirmed by a single band of corresponding molecular weight revealed by agarose gel electrophoresis. The concentration of all target primers was 100 nM, and the CYC primer was 200 nM. 

### Western Blot

One pup from each HFD and chow dam was sacrificed at P15, and the hypothalami were excised and the PVN micro-dissected as indicated above (n=3 or 4). One embryo from each HFD and chow dam was sacrificed at E19, and whole hypothalamus was excised. Tissue was lysed in RIPA lysis buffer and homogenized manually using a mortar and pestle. A Lowry assay was performed to determine the total protein in each sample. Afterwards, the homogenate was concentrated using a protein concentrator (Millipore, Billerica, MA). Since commercially available antibodies specifically against phosphorylated and ubiquitin-TEF were unavailable, TEF was purified from whole protein samples through immunoprecipitation with rabbit anti-TEF-1 antibody (Abcam, Cambridge, MA) and Protein A/G beads (Santa Cruz Biotechnology, Santa Cruz, CA) to determine the relative levels of phosphorylated TEF and ubiquitin-tagged TEF from total TEF protein. A total of 5 µg of prepared protein samples or 5 µL of immunoprecipitated samples were then subjected to SDS-PAGE using a Biorad Tetra Cell (Biorad Inc., Hercules, CA) at 100 V until bands were at the bottom of the gel and transferred to a nitrocellulose membrane. Blots were blocked overnight in 5% BSA and incubated with one of the following antibodies: rabbit anti-TEF-1 (1:2000, Abcam, Cambridge, MA), mouse anti-p-serine (1:1000, Abcam, Cambridge, MA), rabbit anti-YAP (1:1000, Thermo Scientific, Rockford, IL), rabbit anti-p127-YAP (1:1000, Thermo Scientific, Rockford, IL), or mouse anti-β-actin (Santa Cruz Biotechnology, Santa Cruz, CA), followed by HRP-conjugated goat anti-rabbit or mouse antibody (1:2000, Santa Cruz Biotechnology, Santa Cruz, CA). Blots were visualized with Luminol Reagent (Santa Cruz Biotechnology, Santa Cruz, CA) and developed on Kodak Biomax Light film. Blots were stripped using Restore western blot stripping buffer (Thermo Scientific, Rockford, IL). Since the levels of YAP and TEF were much lower than β-actin controls, the β-actin controls were used only to ensure that the protein concentrations from each sample was the same and there was no difference between chow and HFD groups.

### Immunofluorescence

Hypothalamic neurons were dissociated and plated onto coverslips and, after 3 days in culture, were fixed and processed with primary antibodies for YAP, TEF and/or ENK. Primary neuronal cells were cultured on poly-D-lysine (Sigma-Aldrich, St. Louis, MO) coated, #1.5, 18 mm round glass coverslips (Warner Instruments, Hamden, CT). The cells were gently washed with 1% phosphate buffered saline (Invitrogen, Carlsbad, CA), fixed with 2% paraformaldehyde for 15 min at room temperature, permeabilized using 0.01% triton (Shelton Scientific, Shelton, CT) for 5 min, and then blocked with 1% bovine serum albumin (Sigma-Aldrich, St. Louis, MO) for 30 min. The cells were incubated with either mouse anti-ENK (1:300; Millipore, Billerica, MA), rabbit anti-YAP (1:200; Pierce Antibodies, Rockland, IL), or rabbit anti-TEF (1:100; Pierce Antibodies, Rockland, IL) at 4°C overnight, followed by goat anti-mouse Alexa-fluor-488 or goat anti-rabbit Alexa-fluor-405 (1:400, Invitrogen, Carlsbad, CA) at room temperature for 2 hours. Cells were washed and the coverslips attached to glass slides using Prolong gold anti-fade reagent (Invitrogen, Carlsbad, CA). The cells were imaged using a Zeiss Axioplan 2 microscope (Zeiss, Thornwood, NY). Cells from immunofluorescence experiments were processed at the same time using the same exposure conditions, and the total number of cells, both fluorescent and non-fluorescent, from each condition was manually counted using ImageJ [37], as previously described [9]. All immunofluorescence data were collected and analyzed by blind analysis. The number of double-labeled YAP or TEF with ENK cells was calculated relative to ENK or to total number of cells, but since no differences were found between these two calculations, all co-localization results are represented as relative to ENK. While measurement of nuclear and cytoplasmic fluorescence intensity levels to examine the active and inactive forms of TEF and YAP was not possible because the proteins are generally ubiquitously located throughout the neuron and specific antibodies for neither the active nor inactive protein were available, a further analysis of neurons that contained only nuclear or cytoplasmic fluorescence was performed by manual count. 

### Treatment with siRNA

Hypothalami from 4 embryonic groups were dissociated for neuronal cell culture, and for each group, a total of 8 cultured wells were used with 4 wells each for the knockdown and non-knockdown control. Both TEF siRNA (*forward: 5’-GGAAAAUCAUCUUAUCAGATT-3’; reverse: 5’-UCUGAUAAGAUGAUUUUCCTC-3’*) and YAP siRNA (*forward: 5’- CAAUGAUCAGACAACAACATT-3’; reverse: 5’-UGUUGUUGUCUGAUCAUUGTG-3’*) were customized from Life Technologies (Life Technologies, Grand Island, NY). The TEF, YAP, and scrambled negative control (Life Technologies, Grand Island, NY) siRNA were used at a concentration of 75 pmol / µL to transfect hypothalamic neuronal cultures using Lipofectamine RNAiMAX (Life Technologies, Grand Island, NY) for 48 hours. Knockdown efficiency of siRNAs was confirmed by qRT-PCR analysis. The siRNA itself did not affect gene expression as the non-silencing control yielded no change in TEF, YAP, or ENK expression as compared to untreated controls. 

### Data analysis

Statistical analyses with multiple comparisons were completed using a repeated-measure ANOVA, with a Bonferroni post-hoc test to determine significant differences between the groups. A direct comparison between a pair of groups was made using an unpaired two-tailed Student’s *t*-test. Correlations were made using the Pearson product-moment correlation coefficient. The criterion for statistical significance was *p* < 0.05. 

For qRT-PCR analysis, 1 sample per dam in each group was used, for a total of 6-9 samples per diet group. The relative C_T_ values from the samples for each chow or HFD dam were averaged, and the change in peptide expression from the HFD group was calculated relative to the chow group. For siRNA qRT-PCR analysis, the relative C_T_ values from the knockdown and non-knockdown control were averaged, the change in peptide expression from the siRNA knockdown neurons was calculated relative to non-knockdown controls, and the percent change in peptide expression from the 4 groups was averaged. For western blot analysis, band density was quantified using ImageJ [38], and the relative change in band density of YAP or TEF from the HFD group was compared to chow. For immunofluorescence data, the number of YAP-, TEF-, or ENK-expressing cells was divided by the total number of cells in the sample to determine the percentage of cells expressing each peptide and this was then averaged from images obtained from the 4 dams. For localization of TEF or YAP, the number of cytoplasmic or nuclear stained neurons was counted, and the percentage of these cells relative to the total number of cells was calculated. To measure levels of peptide in fixed neurons, after subtraction of the mean background intensity of a nearby area of comparable size, fluorescence intensity measurements were calculated as the mean intensity within the region multiplied by its area, in arbitrary units (a.u.). In all cases, exposure conditions within an experiment were identical. To determine the mean fluorescence intensity and the number of independent variables or neuronal populations, the data were plotted as a frequency count and fitted to a Gaussian distribution. The number of peaks that resulted in an R^2^ value closest to 1 was used. To determine the goodness of fit, X^2^ values corresponding to *p* < 0.05 were considered significant. The number of fitted distributions to the histogram reflects the number of populations, the peak of each distribution reflects the mean intensity, and the area under the curve reflects the percentage of cells within that distribution. Between 300 and 500 cells were measured for each protein. 

## Results

### Prenatal HFD effects on hypothalamic TEF and YAP expression

**Figure 1:**
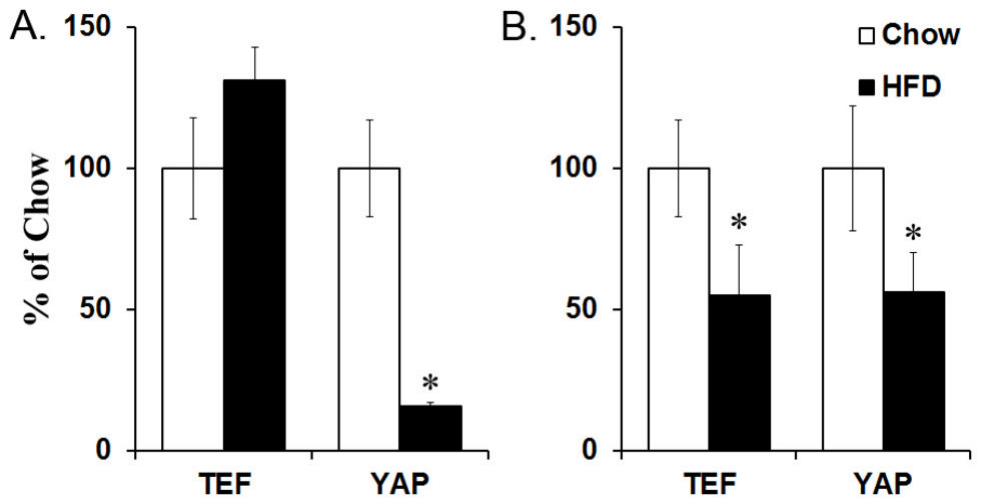
mRNA levels of TEF and YAP. (A) The mRNA was measured via qRT-PCR in the PVN of P15 offspring prenatally exposed to high-fat diet (HFD) or chow, and a statistically significant decrease was found in YAP expression from offspring that were prenatally exposed to high-fat diet (HFD) compared to chow. (B) The mRNA was measured in whole hypothalamic tissue of E19 embryos prenatally exposed to HFD or chow, and a statistically significant decrease was observed for both TEF and YAP expression from HFD embryos compared to chow. The mRNA levels were normalized to cyclophilin and calculated using the CT comparative method. Results are expressed as means ± SEM (n = 6–9). *p < 0.05, compared with chow. YAP = Yes-associated protein; TEF = transcription enhancing factor.

To determine whether prenatal HFD exposure has an effect on the expression of TEF and YAP, mRNA from the PVN of P15 offspring prenatally exposed to a HFD or chow was extracted, and qRT-PCR was performed. An interaction effect between group and mRNA expression was found (*F*(1,19) = 46.13, *p* < 0.001), with a significant decrease in the expression of the co-activator, YAP (-84%; *t*(19) = 6.23, *p* < 0.001), in HFD offspring with no change in TEF expression (*t*(20) = 1.30, *not significant or n.s.*) (Figure 1A). Next, measurements of TEF and YAP mRNA at E19 were taken from whole hypothalamus, since individual regions of the hypothalamus are not fully differentiated at this age. In HFD compared to chow embryos, an interaction effect between group and mRNA expression was also observed (*F*(1,17) = 6.07, *p* < 0.05), this time with a significant decrease in mRNA levels of both TEF (-45%; *t*(17) = 2.47, *p* < 0.05) and YAP (-49%; *t*(17) = 2.12, *p* < 0.05) (Figure 1B) in HFD embryos. These results in P15 offspring and E19 embryos demonstrate that prenatal HFD compared to chow exposure has a generally suppressive effect on the expression of these two transcription factors in the hypothalamus in both embryonic and postnatal offspring, with no change in the expression of TEF in P15 offspring. 

### Prenatal HFD effects on hypothalamic TEF and YAP protein levels

**Figure 2:**
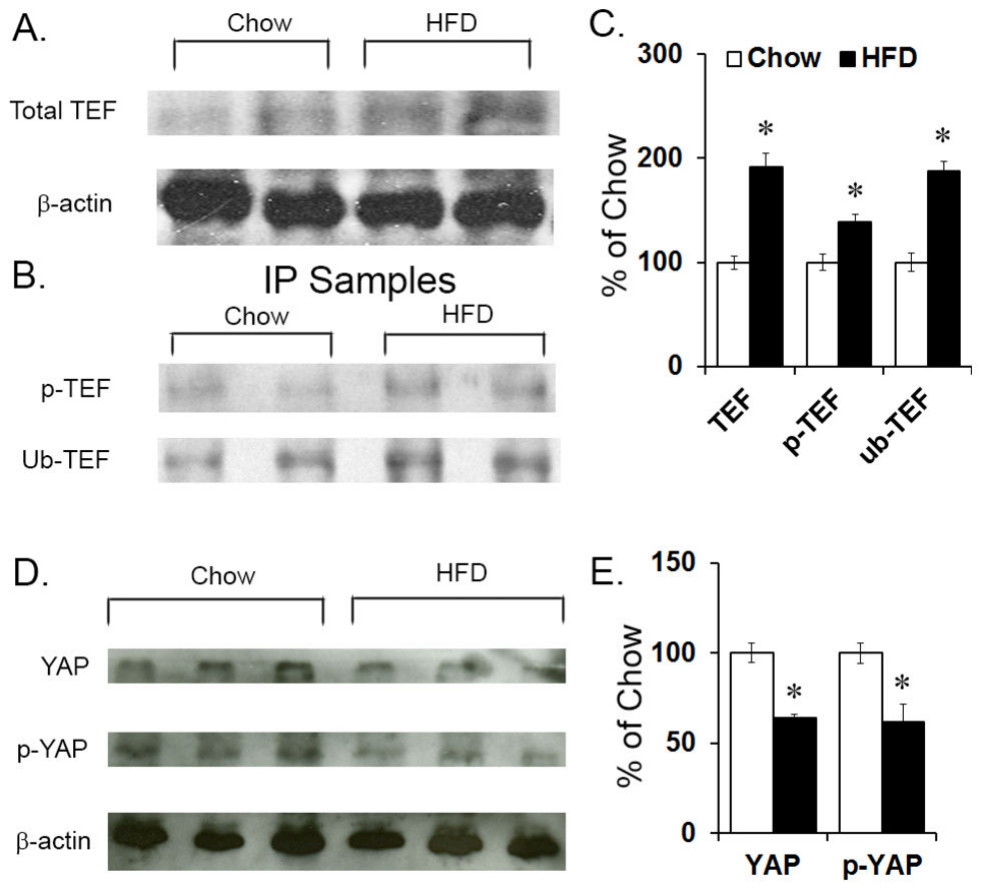
Protein levels in P15 offspring. (A, C) TEF protein level was measured via western blot in the PVN of high-fat diet (HFD) compared to chow P15 offspring, and a statistically significant increase in total TEF was found. (B, C) Further immunoprecipitation (IP) of total TEF protein from the PVN revealed a statistically significant increase in levels of phosphorylated TEF and ubiquitin-tagged TEF. (D, E) YAP protein level was measured via western blot in the PVN of high-fat diet (HFD) compared to chow P15 offspring, and a statistically significant decrease in total YAP and phosphorylated YAP protein was found. Results are expressed as means ± SEM (n = 4) *p < 0.05. The β-actin bands were used to ensure that all samples had the same concentration of protein; they were not used to normalize TEF and YAP bands. The change in TEF and YAP band densities from the HFD group was determined relative to the chow control group. p-TEF = phosphorylated TEF; ub-TEF = ubiquitin-tagged TEF.

**Figure 3:**
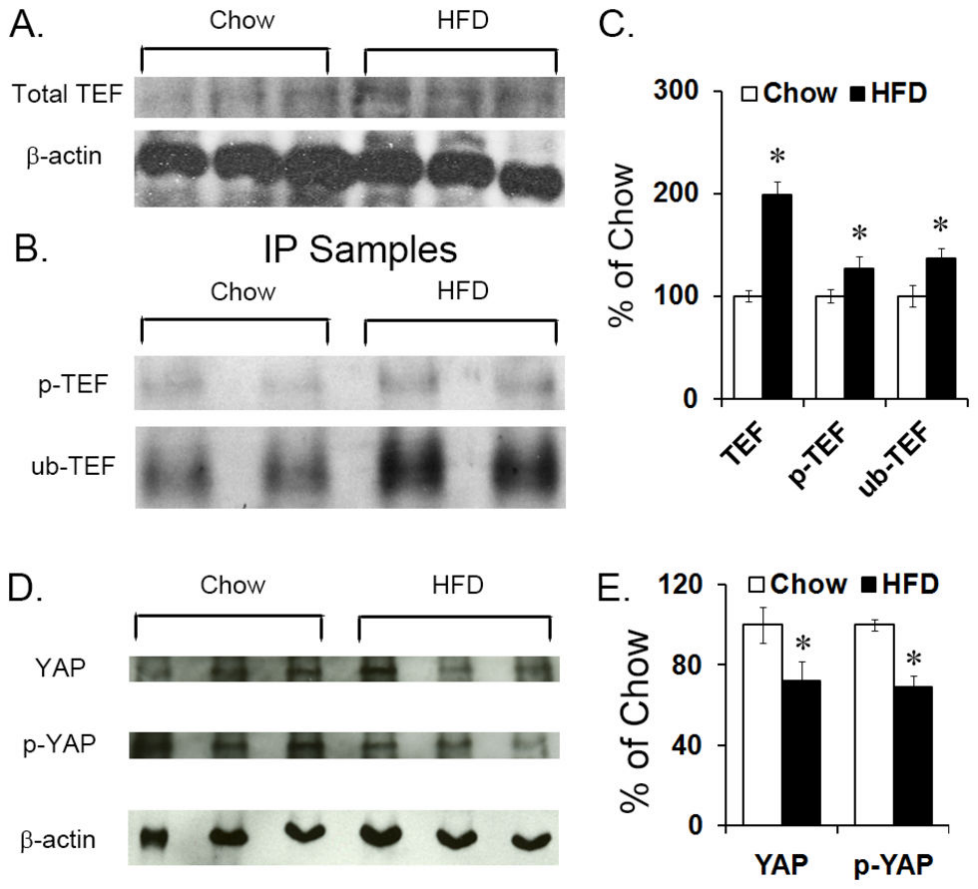
Protein levels in E19 embryos. (A, C) TEF protein level was measured via western blot in the PVN of high-fat diet (HFD) compared to chow E19 embryos, and a statistically significant increase was found for total TEF protein levels in whole hypothalamus. (B, C) Further immunoprecipitation (IP) of total TEF protein from whole hypothalamus revealed a statistically significant increase in levels of phosphorylated TEF and ubiquitin-tagged TEF. (D, E) YAP protein level was measured via western blot in whole hypothalamus of high-fat diet (HFD) compared to chow E19 embryos, and a statistically significant decrease in total YAP and phosphorylated YAP protein was found. Results are expressed as means ± SEM (n = 4). *p < 0.05. The β-actin bands were used to ensure that all samples had the same concentration of protein; they were not used to normalize TEF and YAP bands. The change in TEF and YAP band densities from the HFD group was determined relative to the chow control group.

To determine whether these changes in mRNA were accompanied by changes in protein levels of TEF and YAP and whether these proteins were in their active or inactive forms, western blotting was performed on purified protein extracted from the PVN of P15 offspring, and both the non-phosphorylated (active) and phosphorylated (inactive) forms of TEF and YAP were probed. In the HFD compared to chow, measurements of total TEF showed an increase in P15 offspring exposed to a HFD (+92%; *t*(6) = 11.66, *p* < 0.001) (Figure 2A, C). This unexpected increase in TEF was further probed for the phosphorylated form and whether it was tagged for degradation by ubiquitin. Since commercially available antibodies specifically against phosphorylated and ubiquitin-tagged TEF are unavailable, all forms of TEF were purified from whole protein samples through immunoprecipitation and subsequently probed for the phospho-group or ubiquitin. The results revealed a significant interaction effect between group and protein levels (*F*(1,5) = 45.33, *p* < 0.001) that was attributed to a significant increase in both phosphorylated-TEF (+39%; *t*(4) = 7.14, *p* < 0.05) and ubiquitin-tagged-TEF (+88%; *t*(4) = 11.40, *p* < 0.001; Figure 2B, C). The ratio of phosphorylated to total TEF (1.87:1; *t*(4) = 6.70, *p* < 0.05) and ubiquitin-tagged TEF to total TEF (2.23:1; *t*(4) = 3.31, *p* < 0.05) was also increased in HFD compared to chow offspring. Similar results were obtained in whole hypothalamus from E19 embryos exposed to a HFD, with a significant interaction effect between group and protein levels (*F*(5,20) = 27.42, *p* < 0.001) reflecting an increase in total TEF (+99%; *t*(9) = 2.44, *p* < 0.05; Figure 3A, C) and both phosphorylated (+27%; *t*(4 = 8.90, *p* < 0.05) and ubiquitinated TEF (+37%; *t*(4) = 4.27, *p* < 0.05; Figure 3B, C), with an increase in the ratio of phosphorylated to total TEF (1.16:1; *t*(4) = 5.71, *p* < 0.05) and of ubiquitin-tagged TEF to total TEF (1.16:1; *t*(4) = 2.66, *p* < 0.05) in HFD compared to chow embryos. These measurements of TEF in P15 offspring and E19 embryos exposed to the HFD suggest that the majority of this transcription factor is in an inactive form, indicating that TEF activity is suppressed by this diet. A similar western analysis of YAP in the PVN from P15 offspring revealed a significant decrease in protein levels, in both total YAP (-36%; *t*(8) = 9.63, *p* < 0.001) and inactive S127 phosphorylated YAP (-38%; *t*(4) = 3.91, *p* < 0.05; Figure 2D, E). Further, in the E19 embryos exposed to the HFD, measurements in whole hypothalamus revealed a significant decrease in both total YAP (-28%; *t*(4) = 3.97, *p* < 0.05) and phosphorylated YAP (-30%; *t*(4) = 9.31, *p* < 0.001; Figure 3D, E). Together, this evidence suggests that both TEF and YAP activity, either through phosphorylation or by decreasing levels and expression, are significantly down-regulated *in utero* as well as postnatally.

### Prenatal HFD effects on the number of hypothalamic TEF- and YAP-expressing neurons in E19 embryos

**Figure 4:**
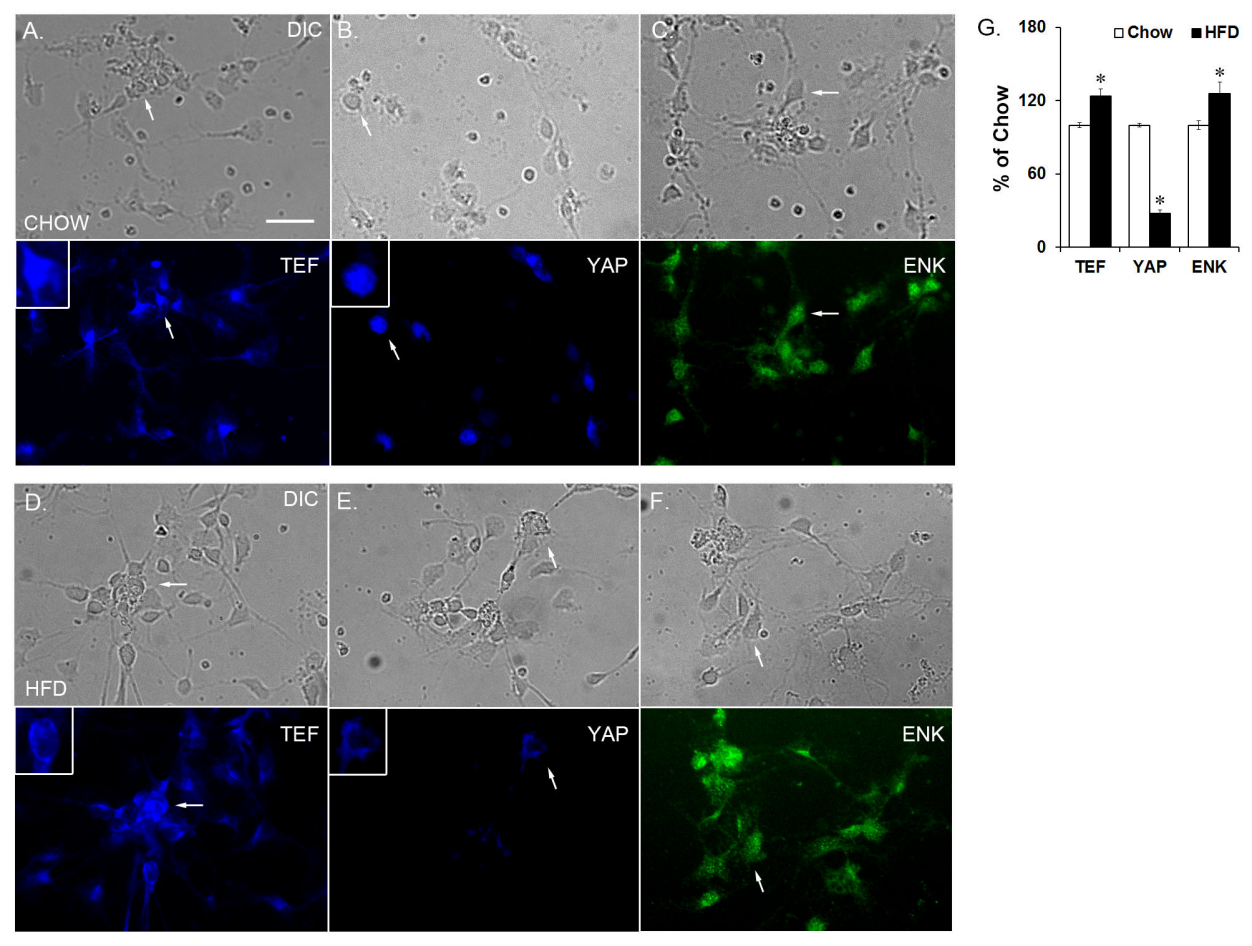
Isolated hypothalamic neurons from E19 embryos. Single-labeling immunocharacterization of dissociated hypothalamic neurons from chow embryos expressing (A) TEF, (B) YAP, or (C) ENK; and from HFD embryos expressing (D) TEF, (E) YAP, or (F) ENK. (G). In the HFD compared to chow embryos, there was a statistically significant decrease in the number of YAP-labeled neurons and an increase in the number of TEF- and ENK-labeled neurons. Top set of images is neurons from chow embryos, bottom set is neurons from HFD embryos. Arrows point to the same cell in the top and bottom image (DIC and fluorescence). Insert shows larger image of neuron with either cytoplasmic or nuclear localization of TEF or YAP. Results are expressed as means ± SEM (n = 4). *p < 0.05, compared with chow. ENK = enkephalin; scale bar is 25 µM.

**Figure 5:**
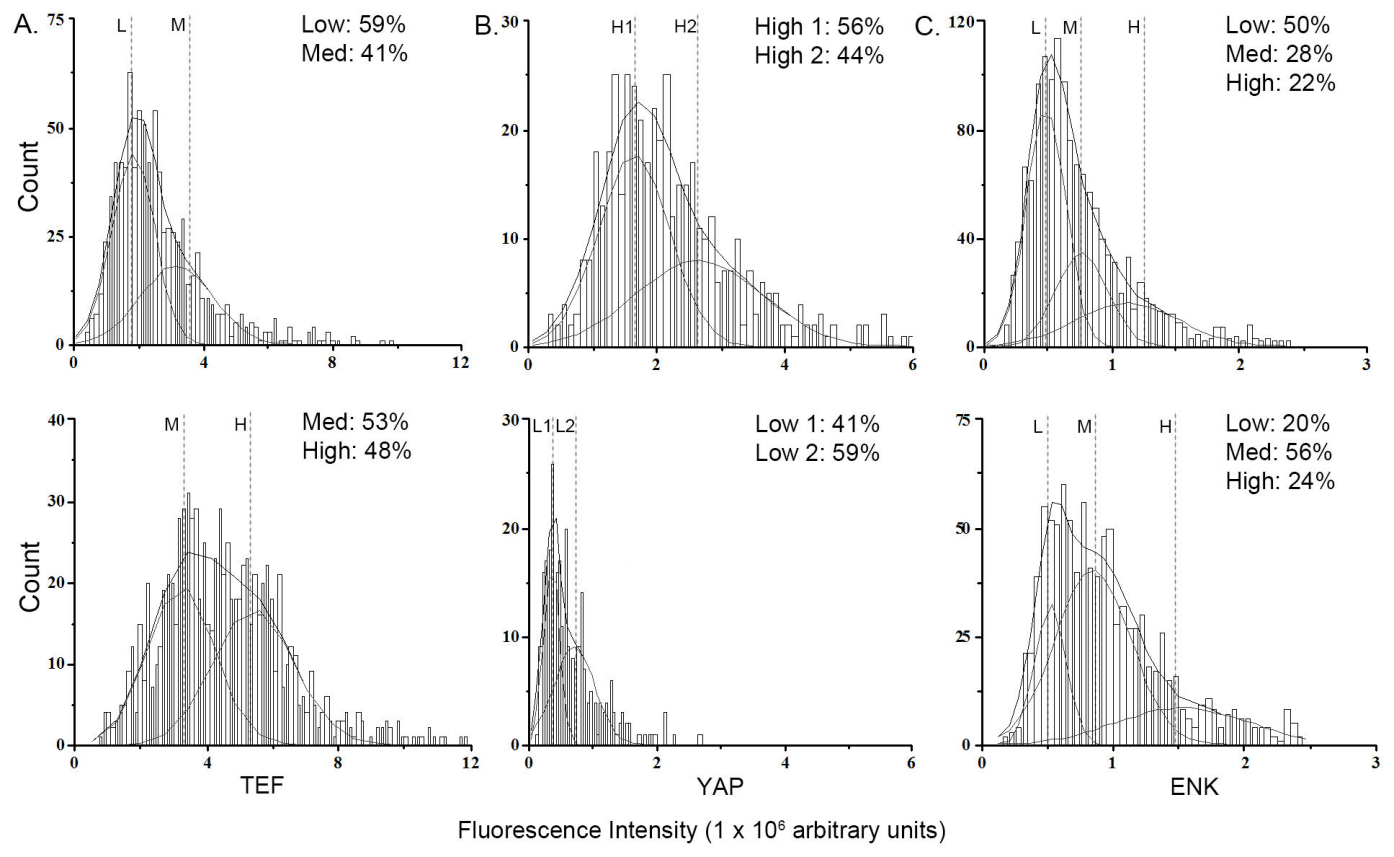
Probability density histogram. The fluorescence intensity for each cell from chow (top) or HFD (bottom) embryos was normalized by subtracting the mean background intensity of a nearby area of comparable size and multiplying the mean intensity within the region by its area, as described in the methods, plotted as a histogram, and fitted to a probability density function. The area under the curve represents the density of cells within each population and is portrayed as a percentage of the total area. (A) Two populations of TEF-expressing neurons were found from chow embryos, arbitrarily assigned as L and M representing low and medium peptide levels, and two were found from HFD embryos, assigned as M and H representing medium and high peptide levels. This shift in fluorescence intensity suggests that HFD increased the levels of TEF in each individual neuron. (B) Two populations of YAP-expressing neurons were found from chow embryos, arbitrarily assigned as H1 and H2 representing high levels of peptides, and two populations were found in neurons from HFD embryos, L1 and L2, representing low levels of peptides. A shift from higher to lower intensity was found in HFD compared with chow embryos, suggesting decreased YAP levels in individual neurons; (C) Three populations of ENK-expressing neurons with the same intensity, L, M and H, were found in both HFD and chow embryos, with a shift in the percentage of neurons exhibiting low intensity to medium and high intensity in the HFD embryos, suggesting increased ENK levels in individual neurons. Goodness of fit was determined based on X^2^ values corresponding to *p* < 0.05.

To determine if these changes in mRNA and protein levels of TEF and YAP are caused by a change specifically in the number of neurons or within individual neurons, dissociated and isolated hypothalamic neurons from E19 embryos were fluorescently labeled (Figure 4A) and manually counted. The fluorescence intensity for each protein from each cell was determined and plotted as a probability density function to determine protein level changes in individual neurons (see Data Analysis in Methods). The HFD compared to chow embryos exhibited an increase in the number of TEF-containing neurons (+24%; *t*(6) = 3.85, *p* < 0.05; Figure 4D). Fluorescence intensity analysis revealed two distinct populations of neurons in chow embryos corresponding to Low (1.82 x 10^6^ a.u.) and Medium (3.15 x 10^6^ a.u.; *p* < 0.001) and two distinct populations of neurons in the HFD embryos corresponding to Medium (3.29 x 10^6^ a.u.) and High (5.51 x 10^6^ a.u.; *p* < 0.001; Figure 5A), reflecting a significant population shift from low and medium intensity in chow neurons to medium and high intensity in HFD neurons. This result demonstrates that the increase in TEF protein levels in HFD embryos reflects an increase in both the density of TEF-containing neurons and the level of TEF within each neuron, consistent with the western blotting results revealing an increase in total TEF protein but also showing this to be mostly in its inactive forms. A similar fluorescence intensity analysis of neurons labeled for YAP in the E19 embryos (Figure 4B) exhibited a significant decrease in the density of YAP-containing neurons (-72%; *t*(6) = 11.14, *p* < 0.001; Figure 4D). Further, fluorescence intensity analysis revealed two distinct populations of neurons in chow embryos exhibiting relatively high intensity, arbitrarily assigned as High 1 and High 2 (1.63 x 10^6^ and 2.65 x 10^6^ a.u.; *p* < 0.05) corresponding to high levels of YAP, and also two populations in HFD embryos that exhibited relatively low intensity, arbitrarily assigned as Low 1 and Low 2 (0.35 x 10^6^ and 0.70 x 10^6^ a.u.; *p* < 0.05; Figure 5B), revealing a non-overlapping shift in fluorescence intensity from the two high levels in chow neurons to the two low levels in HFD neurons. This result, consistent with western blotting analysis, shows that the decrease in YAP protein levels induced by the HFD reflects a reduction in both the density of YAP-containing neurons and the amount of YAP found within each neuron. The non-overlapping fluorescence intensity levels between the HFD and chow neurons further demonstrate a marked difference in the TEF- or YAP-containing neurons as a function of diet. A further count of neurons that had TEF or YAP immunofluorescence completely localized to the cytoplasm showed a significantly higher percentage in the HFD compared to chow neurons (Table 1; Figure 4A, B, D, E, insert). This sequestration in the cytoplasm suggests that these proteins were phosphorylated. 

**Table 1:** Percent of nuclear vs. cytoplasmic TEF or YAP neurons.

	TEF		YAP	
	Nucleus	Cytoplasm	Nucleus	Cytoplasm
Chow	42 ± 0.4%	58 ± 0.4%	78 ± 2.0%	22 ± 2.0%
HFD	34 ± 1.1%	66 ± 1.1%	38 ± 2.6%	62 ± 2.6%

Neurons that contained either nuclear or cytoplasmic fluorescence were manually counted and calculated as a percentage of the sum of both nuclear and cytoplasmic neurons. A higher percentage of neurons that had only cytoplasmic staining for both TEF and YAP was found in neurons from HFD embryos compared to chow. Results are expressed as means ± SEM (n = 4). *p* < 0.05, compared with chow.

### Prenatal HFD effects on the number of hypothalamic ENK-expressing neurons of E19 embryos

To investigate a possible relationship between the HFD-induced change in TEF and YAP and in the number of ENK neurons, our first step was to confirm, using a modified protocol to improve fluorescence labeling, our finding that prenatal HFD increases the density of ENK-expressing neurons in the hypothalamus of E19 embryos [9]. Dissociated hypothalamic neurons were used to specifically measure ENK in neurons, and consistent with our published evidence [9], we observed here with single fluorescence labeling a significant increase in the number of ENK-containing neurons in response to the HFD (+27%; *t*(6) = 2.56, *p* < 0.05; Figure 4C, D). Plotting of immunofluorescence intensity revealed three populations of neurons, corresponding to relative protein levels, in both the HFD and chow neurons, which were arbitrarily assigned as Low (chow: 0.48 x 10^6^ arbitrary units, *a.u.*, HFD: 0.52 x 10^6^ a.u.; *p* < 0.001), Medium (chow: 0.76 x 10^6^ a.u., HFD: 0.84 x 10^6^ a.u.; *p* < 0.001), and High (chow: 1.12 x 10^6^ a.u., HFD: 1.35 x 10^6^ a.u.; *p* < 0.001; Figure 5C). In the HFD compared to chow embryos, the percentage of neurons existing in the high population was much greater, while the percentage of neurons existing in the low population was much lower. These results in E19 embryos demonstrate that prenatal HFD exposure significantly increases the density of neurons in the hypothalamus that contain higher levels of ENK. 

### Effects of prenatal HFD exposure on the co-expression of TEF/YAP with ENK in E19 embryos

**Figure 6:**
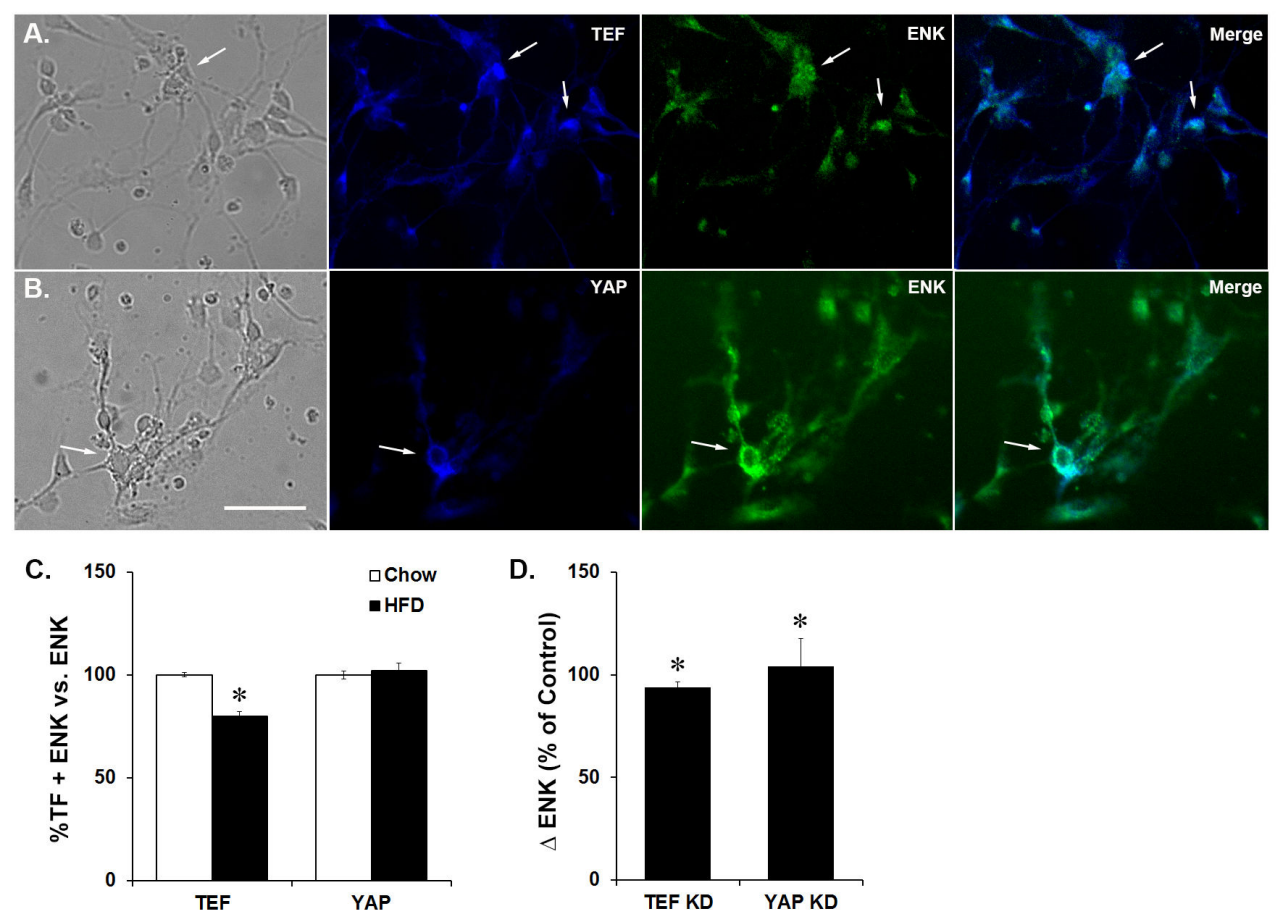
Co-localization of TEF or YAP with ENK. Immunocharacterization of dissociated hypothalamic neurons from E19 embryos co-labeled with (A) TEF and ENK and (B) YAP and ENK. (C) In HFD embryos, there was a significant decrease in the density of co-labeled TEF and ENK neurons but no change in the density of co-labeled YAP and ENK neurons. (D) Knockdown of either TEF or YAP with siRNA results in a significant increase in ENK expression. Representative images of neurons are from HFD embryos. Arrows point to the same cell from images left to right (DIC, fluorescence and merged images). Results are expressed as means ± SEM (n = 4). *p < 0.05, compared with chow. TF = transcription factor; KD = knockdown; scale bar is 25 µM.

There are currently no studies which have examined whether the transcription factors, TEF and YAP, are related to hypothalamic orexigenic peptides. To determine whether TEF or YAP is closely associated with ENK, we first examined whether these transcription factors co-localize with ENK in hypothalamic neurons and then tested whether their co-localization is altered by prenatal HFD exposure. Neurons were fluorescently double-labeled with either TEF and ENK or YAP and ENK. While the percentage of double-labeled neurons was calculated relative to total ENK neurons as well as total cells, no differences were found between these calculations, leading us to present all co-localization results relative only to ENK. In the chow and HFD embryos, both TEF and YAP were found to co-localize with ENK in the hypothalamus (Figure 6A, B), suggesting a close relationship between these transcription factors and the expression of ENK. In comparing the prenatal HFD to chow embryos, the data revealed a significant decrease in the number of double-labeled TEF and ENK neurons in the hypothalamus (-20%; *t*(6) = 10.65, *p* < 0.001; Figure 6C), despite the increase in the density of total TEF neurons. In contrast to TEF, although the density of YAP neurons in HFD embryos was decreased, there was no significant change in the number of double-labeled YAP and ENK neurons (*t*(6) = 1.35, *n.s.*; Figure 6C). These results suggest that, while both TEF and YAP co-localize with and may be involved in controlling ENK expression and neuronal differentiation, TEF may have greater impact in response to the HFD, as reflected by the significantly reduced function of TEF and co-localization of TEF with ENK neurons in HFD-exposed embryos. 

### Effects of TEF or YAP Knockdown on ENK expression in E19 hypothalamic neurons

To determine how decreasing the activity of TEF and YAP affects ENK expression in hypothalamic neurons, expression knockdown studies using small interfering RNA (siRNA) targeted to either TEF or YAP were performed on dissociated hypothalamic neurons from E19 embryos. A significant genetic knockdown efficiency of 45 ± 4% (*t*(6) = 13.21, *p* < 0.001) that was achieved with TEF resulted in a significant increase in ENK expression (96%; *t*(6) = 13.11, *p* < 0.001; Figure 6D) in hypothalamic neurons. This knockdown of TEF correlated significantly with the increase in ENK expression (*r* = 1.00, *p* < 0.001). A significant genetic knockdown efficiency of 72 ± 1% that was achieved with YAP (*t*(6) = 96.76, *p* < 0.001) similarly resulted in a significant increase in ENK expression (+104%; *t*(6) = 104.14, *p* < 0.001; Figure 6D) in hypothalamic neurons. This knockdown also correlated significantly with the increase in ENK (*r* = 1.00, *p* < 0.001). These results strongly link TEF and YAP activity to ENK and show that decreasing these two transcription factors affects neuronal ENK expression.

## Discussion

Prenatal exposure to a HFD has been shown in postnatal offspring to stimulate the expression of hypothalamic orexigenic peptides and increase the density of peptide-expressing neurons in the hypothalamus [4,7,39-42]. The question addressed in this paper is whether these changes in peptide neurons during development are related to changes in specific transcription factors. Focusing on the transcription factor, TEF, with its main transcription factor partner, YAP, this study tested whether prenatal HFD exposure affects the expression and synthesis of these proteins in the hypothalamus and whether these factors may be related to the differentiation and expression of ENK neurons, both at E19 when neuronal differentiation is occurring and at P15 when HFD-induced changes in peptide expression are evident and known to be sustained into adulthood. The results obtained in this study showed that both TEF and YAP are more inactive in embryos and postnatal offspring prenatally exposed to a HFD and that this inactivity, by decreasing expression or increasing the phosphorylated state, increases the expression of ENK, and may be associated with diet-induced changes in the development of hypothalamic ENK neurons. 

### Effects of Prenatal HFD on YAP function

Our studies show in the hypothalamus of E19 embryos and the PVN of P15 offspring that prenatal HFD exposure decreases YAP expression and protein levels, the density of YAP-expressing neurons, and the levels of YAP within each neuron. They also reveal an increase in the percentage of neurons that localize YAP in the cytoplasm, suggesting that YAP was phosphorylated. These findings clearly demonstrate that prenatal HFD suppresses YAP activity towards the end of the gestational period, an effect that continues postnatally. While the main partner of YAP is TEF, YAP can also associate with other factors, suggesting that decreasing YAP levels may also reduce its association with other partner transcription factors, causing a reduction in transcriptional activity [13,43,44].

### Effects of Prenatal HFD on TEF function

The expression of TEF was also found to be reduced in E19 embryos prenatally exposed to HFD. While the HFD increased total protein levels of TEF, density of TEF-expressing neurons, and levels of TEF within each neuron in the E19 embryo and P15 offspring, further analyses showed that the ratio of the inactive phosphorylated and ubiquitinated forms to total TEF protein was significantly higher in HFD neurons. In addition, there was a significantly greater percentage of neurons that localized TEF in the cytoplasm, suggesting that TEF was in the phosphorylated form and its activity suppressed by prenatal HFD exposure. Along with the decrease in YAP, this finding indicates that the association between TEF and YAP was significantly down-regulated. Since TEF also has other binding partners, it has been suggested that high levels of TEF causes competition for association with other transcription factors that may further suppress TEF activity [13,14,45,46]. 

### Relationship between TEF and YAP with ENK in hypothalamic neurons

Prenatal HFD exposure affects the expression and hypothalamic development of ENK neurons in offspring at various embryonic and postnatal ages [4,9]. Through genetic profiling, changes in TEF or YAP activity have been shown to affect the expression of many neurotransmitters and neuropeptides [25] but have never been linked to hypothalamic orexigenic peptide-expressing neurons or to be affected by prenatal HFD exposure. The present findings show significant co-localization of TEF and YAP with ENK in both chow and HFD hypothalamic neurons, a decrease in number of neurons that co-localize TEF and ENK after prenatal HFD exposure, and an increase in ENK expression after knocking down of either TEF or YAP in hypothalamic neurons. These results strongly suggest that these transcription factors are affected by prenatal HFD exposure and are involved in controlling ENK-expressing neurons. While there was no HFD-induced change in the number of neurons that contained both YAP and ENK, the decrease in levels of YAP suggests that there may be a decreased association with TEF, resulting in decreased activity of the associated TEF and YAP complex, and that this may contribute to the increase in ENK expression seen in YAP knockdown neurons. Although both TEF and YAP have other binding partners [43,44,46] and are involved in different signaling cascades, many of which are currently unknown [47-50], the finding that knocking down either transcription factor results in a similar increase in ENK expression further suggests that these specific transcription factors function together to produce the effects seen during prenatal HFD exposure. While this study did not look at other peptide neurons, the fact that TEF and YAP exist in neurons other than those containing ENK suggests that they are likely to affect other neuronal phenotypes. 

### Role of TEF and YAP in the differentiation of peptide neurons

Prenatal exposure to a HFD has been shown to increase neurogenesis and the number of neurons expressing orexigenic neuropeptides in multiple hypothalamic nuclei at various ages, ranging from E14 to P70 [4,7,39-42]. A possible involvement of TEF and YAP in this process is suggested by a few studies in developing embryos showing the suppression of TEF and YAP, either through phosphorylation or repression of expression, to have a stimulatory effect on neuronal differentiation [17,19,24]. Our results demonstrate that prenatal HFD exposure causes increased inactivity of both TEF and YAP and relates this inactivity to an increase in ENK expression. These findings, revealing reduced activity of TEF and YAP in embryos at E19 and also postnatally at P15, are consistent with the timeline showing prenatal HFD exposure to increase the number of peptide-expressing neuroprogenitor populations at E18/E19, which leads to an increase in the number of functional peptide-expressing neurons postnatally, at P15, [8,9,51]. This suggests that the effect of prenatal HFD on TEF and YAP is sustained in the postnatal period and perhaps into adulthood. While comparing only two developmental ages, this study provides initial evidence to support the idea that dietary fat exposure during gestation can affect hypothalamic levels and activity of these transcription factors throughout development and that this effect may alter the differentiation and possibly proliferation of hypothalamic peptide-expressing neurons. Analyses of additional developmental time points and hypothalamic regions will be helpful in further assessing the involvement of TEF and YAP in this process. 

## Conclusions

This study, looking at the involvement of transcription factors in the prenatal programming of offspring exposed to an imbalanced diet, demonstrates that prenatal HFD exposure suppresses the transcription factor, TEF, and co-activator, YAP, in both embryos and postnatal offspring. These transcription factors are known to control organ growth during early development, with dysfunction of either protein found to deregulate and alter organ size development [52-55], and they may be related to the changes in organ size and growth seen in offspring that are prenatally exposed to HFD [56-59], suggesting much broader functions during development. The findings of the present study, showing these transcription factors to both co-exist and affect ENK and be inversely related in hypothalamic neurons, underscore their functions during development, and provide the first evidence that they may be involved in controlling the development of neurons expressing ENK and possibly other neuropeptides or neurotransmitters. Through this relationship to neurochemical systems in the brain, the suppressive effects of HFD exposure on TEF and YAP activity may have a variety of detrimental consequences, affecting both the behavioral and physiological traits in the offspring.
